# Cognitive‐Behavioral Therapy for Health Anxiety and Heart‐Focused Anxiety in Adult Congenital Heart Disease—A Single Case Experiment

**DOI:** 10.1002/ccr3.73293

**Published:** 2026-08-07

**Authors:** Sean Hill, Kayleigh Caffyn, Sarah Scott

**Affiliations:** ^1^ Department of Experimental Psychology University of Oxford Oxford UK; ^2^ Buckinghamshire Healthcare NHS Trust Aylesbury UK; ^3^ Oxford University Hospitals NHS Foundation Trust Oxford UK

**Keywords:** adult congenital heart disease, cardiac anxiety, case report, cognitive‐behavioural therapy, health anxiety

## Abstract

Health anxiety poses unique challenges in long‐term conditions requiring symptom monitoring to prevent deterioration. In ACHD, the nature of health anxiety is poorly understood, and treatment must support health management without reinforcing anxiety. This case demonstrates reduced heart‐focused anxiety and health anxiety in a 25‐year‐old female with ACHD following CBT.

## 1 Introduction

### 1.1 Adult Congenital Heart Disease

Congenital heart disease refers to structural heart or cardiac vessel abnormalities present from birth. It affects approximately 8 per 1000 live births, with around two‐thirds requiring lifelong care [1]. Advancements in diagnosis and treatments have dramatically improved survival rates into adulthood, from approximately 20% to 90% over the last 50 years [2].

Management of adult congenital heart disease (ACHD) involves regular monitoring and in some cases, repeat surgical interventions. Living with ACHD can considerably burden patients' quality of life [3], with chronic fatigue, exercise intolerance, concerns about employment, relationships, and family planning challenges being common [4]. Owing in part to these burdens, anxiety, depression, and substance use disorders are overrepresented in ACHD populations [5, 6].

### 1.2 Health Anxiety and Heart‐Focused Anxiety

The International Classification of Diseases‐11 (ICD‐11; World Health Organization, 2022) defines health anxiety as an intense preoccupation with physical health and fear of experiencing serious illness. Heart‐focused anxiety (HFA [7]), also described as cardiac anxiety [8] or cardiophobia [9], is a form of health anxiety related to cardiac health and sensations. Around one third of cardiac outpatients experience clinically significant health anxiety [10] and HFA [11], with prevalence increasing with older age [12]. HFA has been associated with lower quality of life and rehabilitation engagement [11, 13].

Health‐related anxiety, as well as HFA, is also a common longitudinal outcome of ACHD [14, 15]. Health anxiety and HFA are readily associated with poorer quality of life and treatment engagement within ACHD [16, 17].

### 1.3 The Present Report

Despite recognition of ACHD patients' psychological challenges [18], no studies have examined psychological interventions for health anxiety and HFA in this population. This report examines the application of a manualised CBT intervention for health anxiety in an individual with ACHD, incorporating repeated measurement of HFA. The primary hypothesis is that health anxiety and HFA will decrease over the intervention. The secondary hypothesis is that HFA would reduce to a lesser degree than health anxiety, as the specificity of HFA would indicate it is not specifically adapted for by a standard health anxiety protocol. These hypotheses would together provide important evidence as to the utility of current health anxiety treatments for cardiac patients, including ACHD.

## 2 Case History and Examination

### 2.1 Relevant History

Poppy (pseudonym) was a 25‐year‐old female who identified as White British. She lived with her partner (male, aged 27) and two sons, aged two and four.

#### 2.1.1 Congenital Heart Defect

Poppy was born with severe pulmonary stenosis, a congenital heart defect characterized by narrowing of the pulmonary valve, restricting blood flow to the lungs and straining the right ventricle. Untreated, it can lead to right ventricular hypertrophy or heart failure. The standard intervention for severe cases is balloon valvuloplasty, a surgery to widen the valve by catheterisation [19]. Despite a “severe” diagnosis, Poppy reported relatively preserved physical functioning during childhood. She participated in sport and physical activity without marked fatigue or exercise intolerance. However, she recalled frequently being cautioned about participation due to her condition and discouraged from engaging in sports.

#### 2.1.2 Heart‐Focused Anxiety: “Fear of Dying”

Poppy reported never knowing life without a heart condition or HFA. Poppy recalled being told from approximately age 7 that she would eventually require a balloon valvuloplasty. Her need for surgery was monitored at annual cardiac reviews: “I was waiting for the surgery my entire life”. Her mother was highly preoccupied with Poppy's condition, regularly stating “If you were to die, I would kill myself”. This instilled a sense of responsibility to remain healthy, believing her survival protected her family.

From approximately age 7, Poppy reported becoming increasingly vigilant about her health. She deliberately engaged in exercise to protect her heart, attempted to maintain a healthy diet, attended annual cardiac appointments, and remained highly attentive to her breathing and heart rate. During adolescence, she avoided alcohol, smoking, and tattoos as she believed these could worsen her condition. She described a sense of social isolation and feeling different from her peers due to these efforts.

#### 2.1.3 Significant Health Events

At age 16, Poppy unexpectedly required emergency eye surgery following a routine examination that revealed retinal deterioration, which, without surgery, would have resulted in blindness. This event generated a sense that sudden health emergencies could happen at any time, and not only within cardiac health.

At age 18, Poppy experienced a miscarriage, which she was informed was due to poor fetal blood supply. Poppy interpreted this as another example of her body failing her, and began what she described as a *“self‐destruct mode”*, wherein she rebelled against her lifelong cardiac vigilance: this involved alcohol, smoking and getting multiple tattoos over the course of a few months. Shortly afterward, (aged 19) a cardiac review revealed deterioration requiring balloon valvuloplasty. Poppy believed her rebellious behaviors triggered the need for surgery. The surgery was successful, following which Poppy remained under lifelong ACHD follow‐up with no further medical events directly pertaining to her cardiac condition.

At age 19, Poppy became pregnant and successfully delivered her first child. During her second pregnancy, aged 22, concerns about fetal health were raised and she was invited to consider abortion. She declined, stating she would love her child regardless. The delivery was complicated and required the baby to spend 7 days in neonatal intensive care. Poppy remained at the hospital throughout, “fully expecting him to die”. Unexpectedly, the baby made a full recovery and developed no health abnormalities.

Postpartum, (age 23 onwards) Poppy's health anxiety intensified, particularly around her parental responsibilities and perceived need to maintain her health to protect her children. The traumatic birth led her health anxiety to become vicarious to her children's health, developing catastrophic misinterpretations when they exhibited illness symptoms. At age 25, during a routine ACHD clinic with her cardiologist, Poppy stated she was becoming increasingly withdrawn due to “fear of dying”, and felt unable to engage in meaningful activities. The cardiologist offered a referral to the ACHD Psychology team, which Poppy accepted.

### 2.2 Presenting Difficulties

A Trainee Clinical Psychologist contacted Poppy 2 days later and offered a course of CBT. Psychometric measures were sent to Poppy (Section 3.3) on a weekly basis, starting 3 weeks prior to treatment and throughout. At referral, Poppy exhibited significant health anxiety. For example, she would interpret any physical exertion as a potential sign of a cardiac event. Additionally, she experienced vicarious health anxiety concerning her children's health. For example, if one of them coughed or sneezed, Poppy would become fixated on the possibility of a medical emergency.

Her worries led Poppy to avoid activities; she felt unable to walk her children to preschool, fearing scenarios such as a car skidding off the road and killing them. As a result, her partner handled many routine tasks, which left Poppy feeling she was unable to be a good mother.

## 3 Method

### 3.1 Health Anxiety and Differential Diagnoses

At assessment, a health anxiety model was deemed the best framework for understanding Poppy's difficulties, as her preoccupations and safety‐seeking behaviors primarily related to preserving health (Section 4). When considering alternative hypotheses, generalized anxiety disorder (GAD) was considered as feared stimuli sometimes generalized outside of health (e.g., worrying about being hit by a car). However, a GAD formulation was not deemed the best fit, considering health‐specific cognitive‐behavioral patterns (selective attention to bodily sensations, catastrophic misrepresentations and safety‐seeking behaviors related to health) were central to Poppy's anxiety.

### 3.2 Treatment Goals

Poppy wanted to engage in *“normal mum”* activities without overwhelming fear, such as taking her children to school, creating meaningful memories with them, and reducing how her anxiety interfered with parental responsibilities. Collaboratively, the following goals were set: (1) reduce depression, anxiety, health anxiety, and HFA; (2) reduce avoiding meaningful activities due to “fear of dying”.

### 3.3 Psychometric Assessment

The **Hospital Anxiety and Depression Scale** (HADS [20]) is a 16‐item self‐report questionnaire assessing anxiety (items 1–8) and depression (items 9–16) symptoms in hospital outpatients. Higher scores on 4‐point Likert scales indicate greater anxiety or depression. The scale shows strong reliability for depression (α = 0.93) and anxiety (α = 0.90) [21] and good convergent and concurrent validity [22].

The **Short Health Anxiety Inventory** (SHAI [23]) is a 14‐item self‐report questionnaire assessing health anxiety symptoms. This is an abbreviated form of the Health Anxiety Inventory‐18, removing items concerning a hypothetical illness, to make it appropriate for those with existing health conditions. It shows convergent, discriminant, and criterion‐related validity [23] and strong internal consistency (α = 0.85–0.94; [24]).

The **Cardiac Anxiety Questionnaire** (CAQ [25]) is an 18‐item measure of heart‐focused anxiety, with higher scores on 5‐point Likert scales indicating greater heart‐focused anxiety. The measure shows good consistency and test–retest reliability for cardiac populations [26].

### 3.4 Single Case Experimental Design

To investigate the secondary hypothesis, a single case experiment using A‐B design [27] was used to measure CAQ and SHAI pre‐treatment (Phase A) and during treatment (Phase B). Visual inspection was deemed appropriate to assess change between phases, in line with recommendations from Park et al. [28] to collect multiple data points and monitor for external factors that might influence the outcome.

### 3.5 CBT Intervention

The intervention consisted of 18 weekly meetings via secure remote video conferencing. The intervention followed Salkovskis et al.'s [29] protocol for treatment of health anxiety using CBT. This protocol involves collaborative development of a formulation, identification and modification of safety‐seeking behaviors, and behavioral experiments designed to test feared predictions and develop alternative explanations of symptoms.

#### 3.5.1 Collaborative Guided Discovery and Engagement With the Model

Session 1–3 focused on socializing Poppy to the treatment model. Recent and historical examples of how Poppy's thoughts and behaviors contributed to avoidance of meaningful activities were used to develop formulations throughout treatment. Sessions 1–5 involved psychoeducation about how behaviors intended to reduce distress perpetuated the problem. Psychoeducation was tailored to Poppy's experiences and integrated into all sessions.

#### 3.5.2 Cognitive Interventions

*Thought and behavior records*: Poppy recorded times she (1) experienced a ‘fear of dying’; (2) engaged in safety‐seeking behaviors; (3) practiced the interventions. Records were discussed in‐session throughout treatment to refine formulations and apply strategies to novel situations.

*Cognitive restructuring*: Alternative interpretations of situations evoking ‘fear of dying’ and safety‐seeking behaviors were explored. Methods included ‘Theory A/Theory B' (e.g., ‘I am about to die vs. ‘I am worried because I value my responsibilities') and responsibility pies (e.g., listing out all reasons why anxiety‐provoking situations can occur, then reappraising by dividing responsibility between reasons).

#### 3.5.3 Behavioral Interventions

*Grounding and relaxation*: To help regulate emotions and facilitate engagement with other interventions, guided imagery‐based grounding and relaxation exercises were practiced, one per session for three sessions.

*Cognitive defusion*: Poppy noticed that thoughts about health (e.g., emerging from watching TV, feeling a sensation, hearing her children cough) would lead to rumination, anxiety and belief of imminent catastrophes. To disrupt these cycles, cognitive defusion (adapted for standard CBT; [30]) was introduced between sessions 7–10 and practiced in various out‐of‐session situations throughout the remainder of treatment.

*Behavioral experiments*: Using the Theory A/Theory B paradigm, belief‐testing exercises were designed to challenge Poppy's catastrophic predictions. These involved engaging in tasks she feared would result in harm or death. Later sessions focused on applying these principles to personally meaningful situations, including parenting responsibilities, applying for a new job, and going on holiday.

## 4 Results

### 4.1 Cross‐Sectional Formulation

Poppy's shared examples coalesced to two types of scenario: (1) anticipating threats before leaving the house, believing these could result in her or her children's death; (2) ruminating on health‐related thoughts, leading to catastrophizing.

The first scenario commonly occurred before outings, especially before taking her children somewhere. Poppy would visualize potential threats, overestimate their severity and likelihood, and predict fatal outcomes. To manage her anxiety, she often delegated these tasks to her partner, later feeling “guilty and stupid” when they returned safely. The second scenario occurred several times per week, typically triggered by stimuli related to health or responsibility (e.g., news reports, experiencing pain or her children coughing), which initiated rumination and hypervigilance about health threats and belief of imminent catastrophes.

**FIGURE 1:**
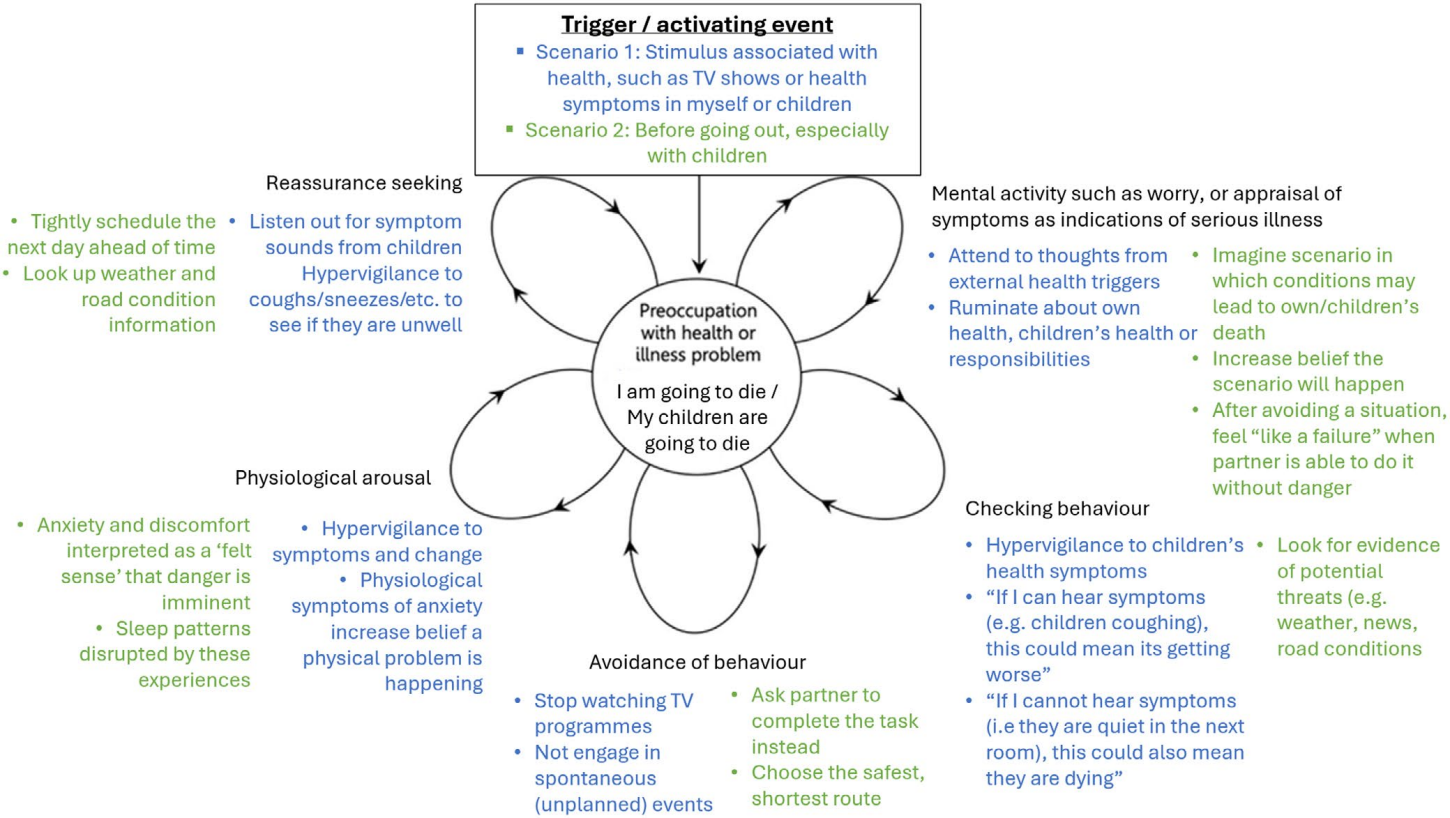
Vicious flower maintenance formulation of Poppy's health anxiety, adapted from Salkovskis et al. [29].

Poppy's interpretation and responses to these scenarios appeared to constitute maintenance of health anxiety and “fear of dying” through hypervigilance, checking, reassurance‐seeking, mental activity and avoidance behaviors: this model was co‐constructed collaboratively, presented in Figure 1.

### 4.2 Longitudinal Formulation

**FIGURE 2:**
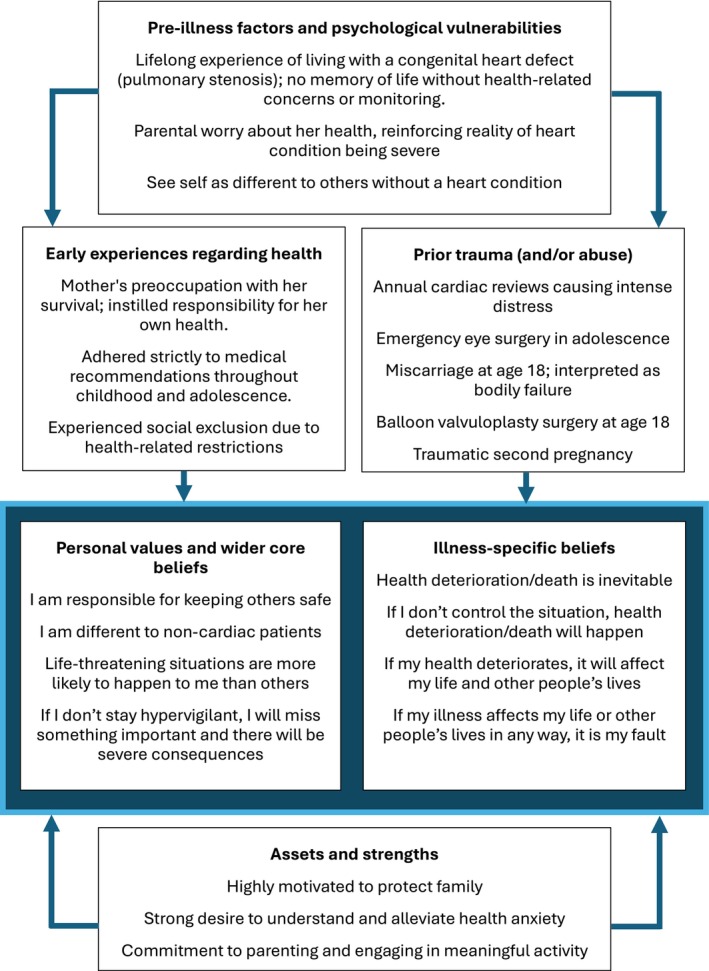
Longitudinal formulation of Poppy's experience of ACHD, adapted from White [31].

Following White's [31] recommendations for adapting therapies to health contexts, a longitudinal formulation captured how Poppy's ACHD‐related beliefs developed over time. This model incorporates (1) pre‐illness factors; (2) early life experiences; (3) personal values and beliefs; (4) prior trauma or abuse; (5) strengths and assets. The longitudinal formulation is presented in Figure 2.

### 4.3 Outcome Measurement

**FIGURE 3:**
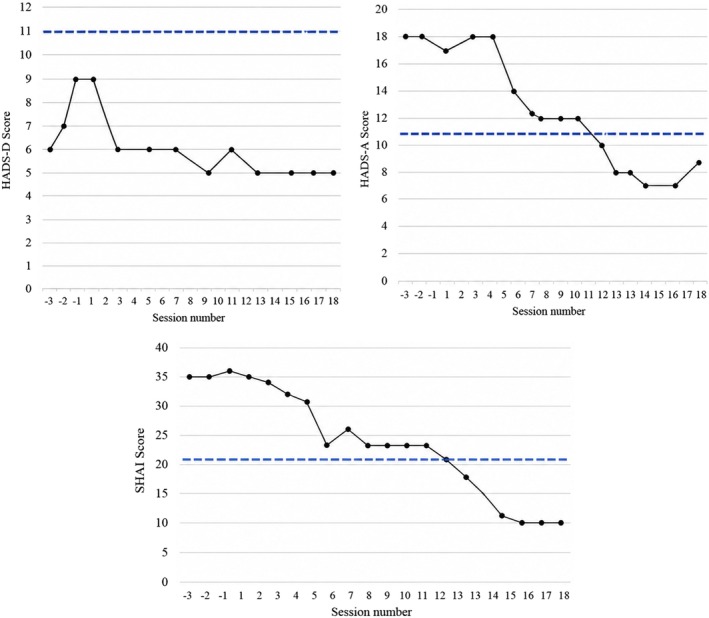
Poppy's HADS‐D, HADS‐A and SHAI scores 3 weeks prior and throughout treatment. A dashed line indicates clinical ‘caseness’ thresholds for each measure (HADS‐A = 11, HADS‐D = 11, SHAI = 22).

Over treatment, Poppy's HADS‐A scores reduced from 18 to 9; HADS‐D scores reduced from 6 to 5; SHAI scores reduced from 35 to 10. Scores are presented in Table 1, and graphically in Figure 3. At 12‐week follow‐up, results were maintained or improved further, with HADS‐A = 6, HADS‐D = 2, and SHAI = 10.

**TABLE 1:** Psychometric scores at the start and end of treatment, follow‐up, and clinical ‘caseness’ thresholds (scores meeting or exceeding validated cut‐offs for clinically significant symptoms).

Measure (construct)	‘Caseness’ threshold	Start of intervention	End of intervention	12‐week follow‐up
HADS‐A (Anxiety)	11	18^a^	9	6
HADS‐D (Depression)	11	6	5	2
SHAI (Health anxiety)	14	35^a^	10	10
CAQ (Heart‐focused anxiety)	N/A	28	20	6

Abbreviations: CAQ, Cardiac Anxiety Questionnaire; HADS‐A, Hospital Anxiety and Depression Scale—Anxiety subscale; HADS‐D, Hospital Anxiety and Depression Scale—Depression subscale; SHAI, Short Health Anxiety Inventory.

^a^ Score meets or exceeds clinical caseness threshold.

### 4.4 Single Case Experiment Results

**FIGURE 4:**
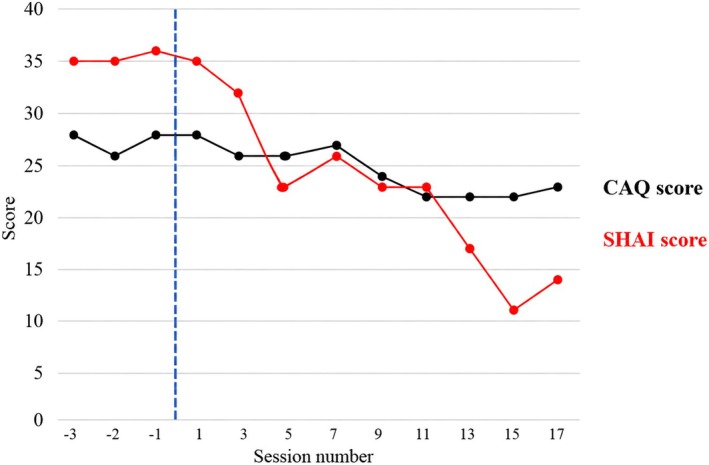
Poppy's SHAI and CAQ scores 3 weeks prior to treatment and every two sessions throughout. A dashed line indicates pre‐intervention (Phase A, left of the line) and intervention (Phase B, right of the line) stages of measurement. The CAQ does not have a caseness threshold, and so no dashed horizontal line is included.

Pre‐intervention, Poppy's CAQ and SHAI scores showed a stable baseline (26–28 and 35–36 respectively). CAQ reduced during the intervention, to 20 at session 17. Visual examination (Figure 4) appears to show a slight but not significant contribution of the intervention to reducing CAQ, and a comparatively greater reduction in SHAI. At 12‐week follow up, Poppy's CAQ scores had reduced further, to 6.

### 4.5 Subjective Response to Treatment

Poppy increasingly recognized how attempts to eliminate uncertainty and catastrophic outcomes maintained her difficulties. She came to understand her “fear of dying” reflected a desire to protect herself and her children rather than evidence of imminent danger. She particularly valued the collaborative, empirical formulation approach, cognitive defusion, and behavioral experiments involving previously avoided activities. Poppy reported engaging in meaningful activities previously avoided, such as taking her children to school and managing routine changes with less anxiety.

## 5 Discussion

The report demonstrates important findings about the effectiveness of CBT for health anxiety in the context of ACHD. Visual inspection of the graphs provides support of the primary hypothesis, with health anxiety, anxiety, and depression scores reducing to below clinical caseness over the intervention. These results were also sustained at 12‐week follow‐up. By contrast, HFA, while reducing slightly, did not appear to reduce notably over the course of the intervention, although it improved markedly at follow‐up. The secondary hypothesis was therefore supported, in that visual inspection indicated a reduction of HFA, but to a lesser extent than health anxiety. However, the addition of the follow‐up scores indicates that HFA did reduce to a notable degree overall, although this did not occur until after the intervention.

At 18 sessions, the intervention was moderately long but within typical treatment duration for CBT for health anxiety [29]. While HFA scores reduced only modestly during the intervention, they demonstrated marked improvement at 12‐week follow‐up, suggesting the benefits for HFA emerged after CBT concluded. This suggests improvements in health anxiety may, by some mechanism, sequentially lead to improvements in HFA. It is possible that adaptations targeting HFA more directly, or modifications tailored to the complexities of ACHD, may be warranted.

### 5.1 Clinical Implications

These findings support that psychological formulations specific to cardiac patients may enhance treatment efficacy. When considering ways to refine CBT for cardiac patients, panic disorder treatments may offer relevant strategies, as panic‐specific strategies often involve a degree of cardiovascular monitoring [32, 33]. Candidate techniques for consideration include idiographic versions of behavioral experiments, interoceptive exposure, and distress tolerance skills aimed at reducing beliefs about physiological arousal, including cardiac sensations.

In addition, the chronic and potentially life‐threatening nature of ACHD likely perpetuates health anxiety and HFA. This case demonstrates the applicability of a longitudinal formulation, incorporating lifelong health experiences, in standard health anxiety treatment. It is notable that longitudinal chronic health formulations are most often conceptualized for older adult cases [34], whereas this case involved a young woman and demonstrates the applicability of longitudinal formulations in this group.

It was noteworthy that HFA observed a delayed improvement trajectory, identified only at follow‐up. A few mechanisms might explain this: for example, reductions in health anxiety and associated avoidance may later allow patients to apply strategies in increasingly meaningful real‐world contexts. This suggests the potential adaptation of standard CBT for health anxiety protocols to place greater emphasis on follow‐up support to reinforce learning post‐intervention, or research examining the trajectories of health anxiety and HFA in cardiac populations.

### 5.2 Study Limitations

Poppy's HADS‐D scores remained below clinical caseness throughout treatment, so it is unclear if similar improvements would occur in cases with higher baseline depression. This limits generalizability, given the high prevalence of depression in ACHD. Additional adaptations, such as behavioral activation, may be needed for those with more severe depression. Secondly, although the CAQ and SHAI are validated for cardiac patients [7, 24, 25], they may not capture the complexities of ACHD patients' lifelong health experiences.

Lastly, although Poppy was able to engage in previously avoided activities, objective functional improvements were not measured. Clinicians may consider the use of functional outcome measurement, such as the Work and Social Adjustment Scale (WSAS; [35]), which is widely used in UK clinical services [36].

## Author Contributions

**Sarah Scott:** supervision, resources, conceptualization, investigation, writing – original draft, methodology, validation, project administration, writing – review and editing. **Sean Hill:** conceptualization, investigation, writing – original draft, methodology, formal analysis, writing – review and editing. **Kayleigh Caffyn:** investigation, writing – original draft, methodology, supervision.

## Funding

The authors have nothing to report.

## Ethics Statement

In accordance with local institutional policy, formal Research Ethics Committee approval was not required for publication of an anonymised single case report. A pseudonym has been used to denote the patient.

## Consent

The person described in the case study has read the submitted version of the manuscript and provided informed written consent for the authors to submit the manuscript for publication. Evidence of consent is available on request.

## Conflicts of Interest

The authors declare no conflicts of interest.

## Data Availability

The data that support the findings of this study are available from the corresponding author upon reasonable request.
